# Cross-talk between intestinal epithelial cells and immune cells in inflammatory bowel disease

**DOI:** 10.1038/srep29783

**Published:** 2016-07-15

**Authors:** Sara Al-Ghadban, Samira Kaissi, Fadia R. Homaidan, Hassan Y. Naim, Marwan E. El-Sabban

**Affiliations:** 1 Department of Anatomy, Cell Biology, and Physiological Sciences, Faculty of Medicine, American university of Beirut, Beirut, Lebanon; 2 Inflammation group-Nature Conservation Center (NCC) for Sustainable Futures, American University of Beirut, Lebanon; 3 Department of Physiological Chemistry, University of Veterinary Medicine Hannover, Hannover, Germany

## Abstract

Inflammatory bowel disease (IBD) involves functional impairment of intestinal epithelial cells (IECs), concomitant with the infiltration of the lamina propria by inflammatory cells. We explored the reciprocal paracrine and direct interaction between human IECs and macrophages (MΦ) in a co-culture system that mimics some aspects of IBD. We investigated the expression of intercellular junctional proteins in cultured IECs under inflammatory conditions and in tissues from IBD patients. IECs establish functional gap junctions with IECs and MΦ, respectively. Connexin (Cx26) and Cx43 expression in cultured IECs is augmented under inflammatory conditions; while, Cx43-associated junctional complexes partners, E-cadherin, ZO-1, and β-catenin expression is decreased. The expression of Cx26 and Cx43 in IBD tissues is redistributed to the basal membrane of IEC, which is associated with decrease in junctional complex proteins’ expression, collagen type IV expression and infiltration of MΦ. These data support the notion that the combination of paracrine and hetero-cellular communication between IECs and MΦs may regulate epithelial cell function through the establishment of junctional complexes between inflammatory cells and IECs, which ultimately contribute to the dys-regulation of intestinal epithelial barrier.

The intestine displays a low-grade physiological inflammation, which is exquisitely regulated^1^. Under pathological conditions such as inflammatory bowel diseases (IBD), the mucosa of the intestine is infiltrated by inflammatory cells, which become situated in close proximity to the epithelial cell layer and thus can play a role in the regulation of its function^2^. In addition to the inflammatory mediators exerting their effect directly or indirectly on epithelial cells, and cell surface adhesion molecules of inflammatory cells (such as selectins and integrins), direct communication between the two cell types *via* gap junction (GJ) might play a role in the regulation of epithelial function. Gap junctions are clusters of intercellular plasma membrane channels, which serve as conduits for intercellular communication that allow passage of ions and low molecular weight metabolites (less than 2 kDa) between the cytosols of two adjacent cells. Gap junctions are composed of members of highly homologous family of proteins known collectively as connexins (Cxs)^3^,^4^. Different connexins can selectively interact with each other to form homomeric, heteromeric, homotypic, and heterotypic channels, which differ in their content and spatial arrangement of connexin subunits and hence permeability of the channels. Gap junction biosynthesis and assembly are strictly regulated and connexins have a short half-life of only a few hours^5^. During their life cycle, Cxs interact with different proteins, including cytoskeletal components such as microtubules, actin, and actin-binding proteins, junctional molecules including adherens junction components such as cadherins, α- and β-catenin, as well as tight junction components such as claudins, occludins and ZO protein. They also interact with enzymes such as kinases and phosphatases and other proteins such as caveolin^6^,^7^,^8^,^9^.

Gap junctional channels have been described in intestinal epithelial cells (IECs) using freeze-etching technique^10^ and detected in the intestine of many species including fish, rabbit^11^, rat^12^,^13^, and human^14^. Intercellular communication between IECs and immune cells has been suggested due to the presence of fenestrations over the villous basal lamina that represent passages or tracks of immune cells^11^,^15^. Gap junction intercellular communication (GJIC) plays an important role in many pathophysiological processes such as neurodegenerative diseases, autoimmune thyroid diseases, acute pancreatitis, cholestasis, diabetes, and glomerulonephritis^16^,^17^,^18^,^19^,^20^. Little is known, however, about the role of GJIC in the inflammation process and vice versa although some reports have suggested that some pro-inflammatory mediators are involved in its regulation^21^,^22^,^23^. In IBD, epithelial cell integrity and function are compromised. We have shown previously that mouse IECs and MΦ establish GJIC^24^,^25^. We have also shown that cytokines such as IL-1, whose levels are increased in the mucosa of IBD patients, mediate their effects on IECs through two distinct lipid metabolic pathways, both of which lead to increased expression of cyclooxygenase-2 enzyme and increased production of Prostaglandin E2 (PGE_2_)^26^,^27^. The overall aim of this study is to explore the nature of the interaction between human IECs and MΦ, to identify the connexin proteins present in human IECs, and to assess their regulation under inflammatory conditions and their potential role in the etiology and pathophysiology of IBD.

## Results

### Expression and functional analysis of connexins in cultured intestinal epithelial cells

**Figure 1:**
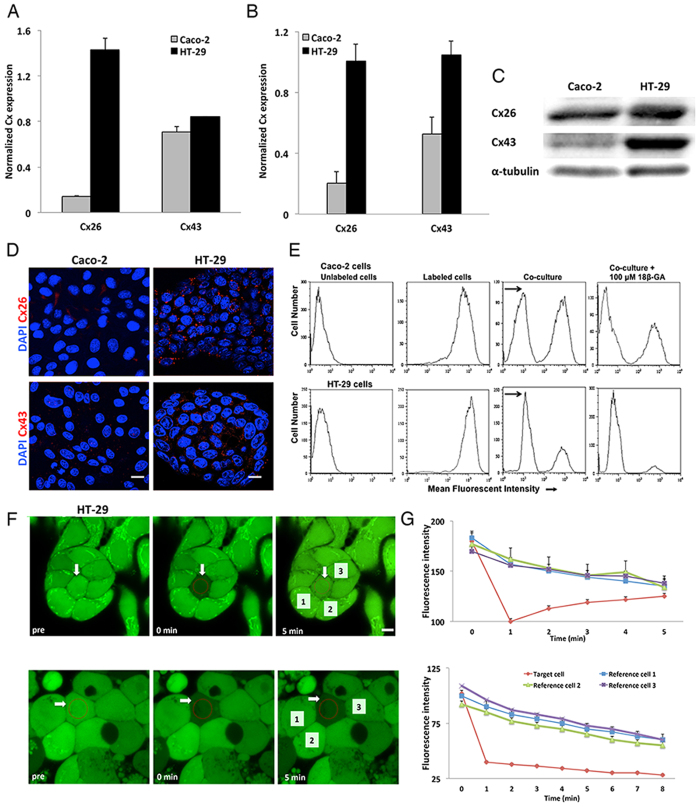
Expression and functionality of connexins in IECs. (**A**) Histogram represents the normalized expression of Cxs to GAPDH assessed by qPCR. (**B**) Densitometry analysis of western blots of Cx expression normalized to GAPDH in IECs. The results are presented as the means ± S.E. of four independent experiments. (**C**) Representative western blot of Cx26 and Cx43 expression in IECs. (**D**) Cellular localization of Cxs, scale bar 10 μm. (**E**) Homo-cellular gap junctional intercellular communication established between IECs, a representative flow cytometry graph where the shift in MFI is shown following co-culture of unlabeled IECs with calcein-labeled IECs; inhibition of this communication is achieved using 100 μM of 18β-GA. (**F**) Fluorescence Recovery After Photo bleaching of HT-29 cells. The white arrow indicates the target cell in untreated cells (upper panel) and in treated cells with 100 μM of 18β-GA (lower panel), scale bar 5 μm. (**G**) Histogram analysis of fluorescent intensity of target cell relative to reference cells of untreated (upper panel) and treated cells (lower panel). Error bars represent the fluorescent intensity of each cell based on several measurements calculated by Zeiss software.

In order to determine which Cxs are involved in the coupling between human IECs, screening for the different Cxs at the transcriptional, translational, and cellular localization levels was performed. Connexin expression was assessed in two human intestinal epithelial cells (IECs): Caco-2, and HT-29. The two cell lines express different levels of Cx26 and Cx43. Caco-2 cells expressed low level of Cx26 transcript but similar level of Cx43 transcript as compared to HT-29 cells (Fig. 1A). These data are supported by western blot analysis showing the expression levels of Cxs in Caco-2 cells and HT-29 cells (Fig. 1B,C). At the cellular localization level, we observed by immunostaining assay the expression and localization of Cx26 and Cx43 in HT-29 cells, but we could not detect Cx expression in Caco-2 cells (Fig. 1D).

To determine the functionality of connexins in forming functional gap junction between IECs, two modalities were conducted: (1) Dye transfer and (2) Fluorescence Recovery After Photo bleaching (FRAP) assays. For dye transfer assay, calcein-labeled IECs were co-cultured with unlabeled IECs at 1:1, 1:2, and 2:1 ratio for predetermined time points. Dye transfer between IECs was evaluated by flow cytometry and mean fluorescence intensity (MFI) was quantified. We observed a shift in fluorescence intensity in unlabeled IECs. The optimal conditions that resulted in this transfer were 2:1 ratio for Caco-2 cells and 1:1 ratio for HT-29 cells. Addition of 100 μM of gap junction–inhibitor, 18β-GA, reduced IECs fluorescence emphasizing that dye transfer occurred through gap junctions (Fig. 1E).

To further confirm the functionality of connexins in IECs, FRAP assay was conducted on HT-29 cells. A single cell within the calcein-loaded monolayer was photo bleached and recovery of fluorescence was recorded immediately after photo bleaching for up to 5 min (Fig. 1F,G). The target cell regained 50% of the initial fluorescence intensity within the first 3 min, and recovered to the same fluorescent level of the neighboring unbleached cells (reference cells) after 5 min. Adding 100 μM of 18β-GA to the bleached cell prevented fluorescence recovery further demonstrating the functionality of the homo cellular GJ between IECs (Fig. 1H,I).

### Expression and functional analysis of connexins in monocyte/macrophage-like THP-1 cell line

**Figure 2:**
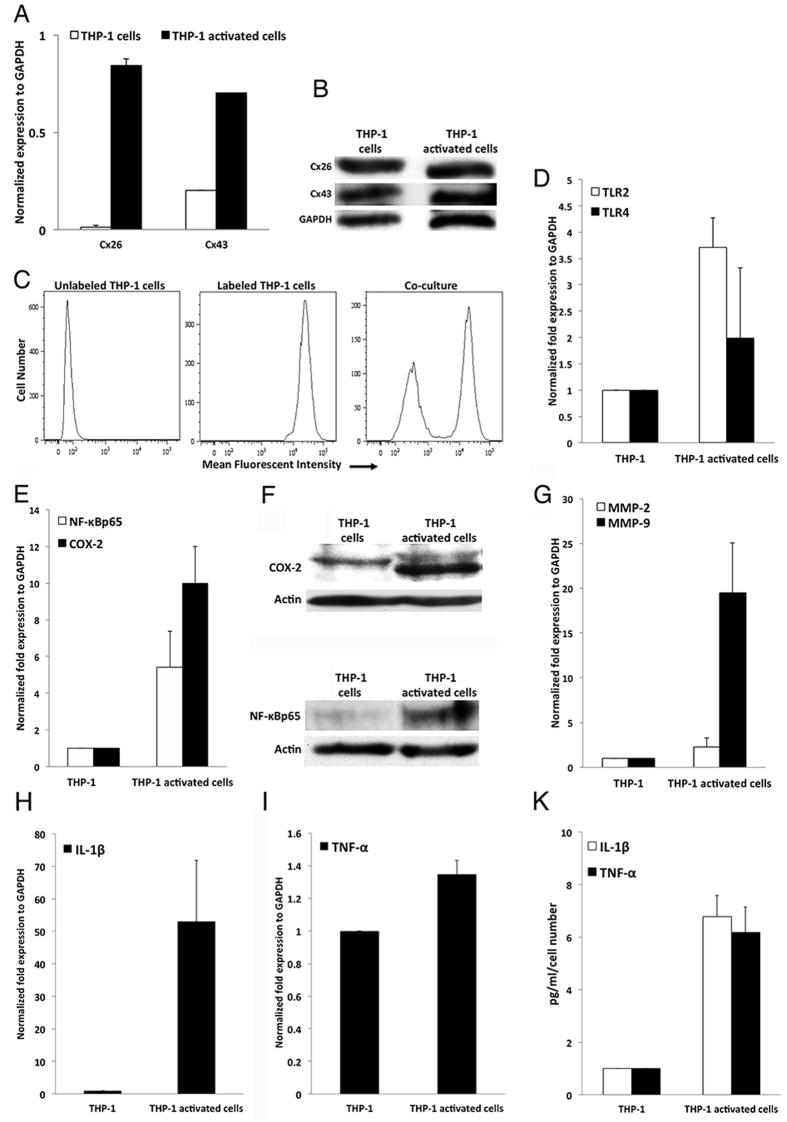
Expression and functionality of connexins in monocytes/macrophages THP-1 cell lines. (**A**) Histogram represents the expression of connexins in THP-1 cells normalized to GAPDH assessed by qPCR. (**B**) Representative western blot of Cx26 and Cx43 expression in IECs. (**C**) Homo-cellular gap junctional intercellular communication established between THP-1 cells. (**D**) Expression of TLR2 and TLR4 assessed by qPCR. Histogram represents the normalized expression to GAPDH. (**E**) Densitometry analysis of western blots of NF-κBp65 and COX-2 expression normalized to actin in THP-1 cells. (**F**) Representative images of western blot of NF-κBp65 and COX-2, respectively. Expression of (**G**) MMP-2 and MMP-9, (**H**) IL-1β, (**I**) TNF-α, as assessed by qPCR. Histogram represents the normalized expression to GAPDH. The results are presented as the means ± S.E. of three independent experiments ((**D**), for TLR2 p = 0.0057; E, for NF-κB p65 p = 0.04, for COX-2 p = 0.008; (**G**) for MMP-9 p = 0.063; (**H**) for IL-1β p = 0.053; (**I**) for TNF-α p = 0.03). (**K**) IL-1β and TNF-α protein level determination by ELISA which was performed per manufacturers’ kit protocols. The results are presented as the means ± S.E. of three independent experiments (for IL-1β, p = 0.012 and for TNF-α, p = 0.015).

To study the interaction between IECs and macrophages, we have used the monocytic cell line THP-1 that has been established as an *in vitro* model for activated macrophages^28^,^29^,^30^. Connexin expression was assessed in the monocytes/macrophages THP-1 cell line at the transcriptional, translational, and functional levels. We showed that THP-1 cells express Cx26 and Cx43 transcript (Fig. 2A) and protein (Fig. 2B). Activation of THP-1 cells by PMA and LPS increased the expression of connexins at the transcriptional level but not at the translational level (Fig. 2A,B). Functionality of the Cxs expressed in THP-1 cells was further assessed by the transfer of calcein dye from unlabeled to labeled THP-1 cells in a co-culture system (Fig. 2C).

Activation of THP-1 cells by PMA and LPS was assessed by qPCR for TLRs expression. We showed that this activation significantly up regulates TLR2 expression by 4-fold (p = 0.0057; Fig. 2D), and insignificantly TLR4 expression by 2-fold (p = 0.17; Fig. 2D) in-activated THP-1 cells as compared to control cells. To further document the activation of THP-1 cells, NF-κB p65 and COX-2 protein levels were determined by western blot (Fig. 2F). A significant 5.5- and 10- fold increase in NF-κB p65 and COX-2 protein expression was detected in activated cells, respectively (p < 0.05; Fig. 2E). Activation of THP-1 cells also induced the expression and secretion of inflammatory cytokines such as TNF-α and IL-1β. We investigated their expression at transcriptional level and showed a significant increase in expression of TNF-α and IL-1β by 1.4- and 50-fold, respectively, in activated THP-1 cells as compared to the controls (p ≤ 0.05; Fig. 2H,I). We then confirmed the data by measuring their protein levels in conditioned media by ELISA. We determined that TNF-α and IL-1β protein levels increased significantly by 6- and 7-fold, respectively, in activated THP-1 cells as compared to control cells (p < 0.05; Fig. 2K). We also investigated the expression of matrix metalloproteinases (MMPs) at the transcriptional level and their zymogenic activity in THP-1 cells. A statistically insignificant 2.5-fold and a 20-fold increase in expression of MMP-2 and MMP-9 was detected in activated THP-1 cells, respectively (p = 0.063; Fig. 2G). The increase in MMP-9 expression at the transcriptional level was accompanied by an increase in its enzymatic activity assessed by gelatin zymography (data not shown).

### Establishment of hetero-cellular communication between IECs and THP-1 cells *in vitro*

Co-culture studies were performed between IECs and macrophages to assess whether hetero-cellular GJIC exists between these cell populations. Dye transfer experiments were conducted with both non-activated (in suspension) and activated THP-1 cells (seeded in six-well plates); in the latter case, IECs were seeded on top of activated THP-1 cells, to mimic the cyto-architecture observed in colon tissue. We have estimated the stoichiometry of infiltrating macrophages into the subepithelial space to be approximately one or two macrophages to one intestinal epithelial cell. This is based on reviewing histological sections of colon tissues, which are displayed on several websites studying IBD cases. For co-culture experiments performed *in vitro*, the ratio of IECs to THP-1 cells was established from data where different ratios of IECs to THP-1 cells (0.5, 1, and 2 and vice versa) and time courses (30 min, 1 h and 2 h) were investigated. The optimal condition, ratio 1:2 of IECs to THP-1 cells, was chosen where maximal dye coupling (MFI) occurred between these two cell types. This is observed by an appreciable shift in fluorescence in the recipient cells, which indicates the transfer of calcein dye from donor labeled cells (IECs) to recipient unlabeled cells (activated THP-1 cells). Further, the experimental procedure was tuned to maximize the intercellular interaction between the two cell types, which increased confidence in the observable extent of change under different culture conditions.

**Figure 3:**
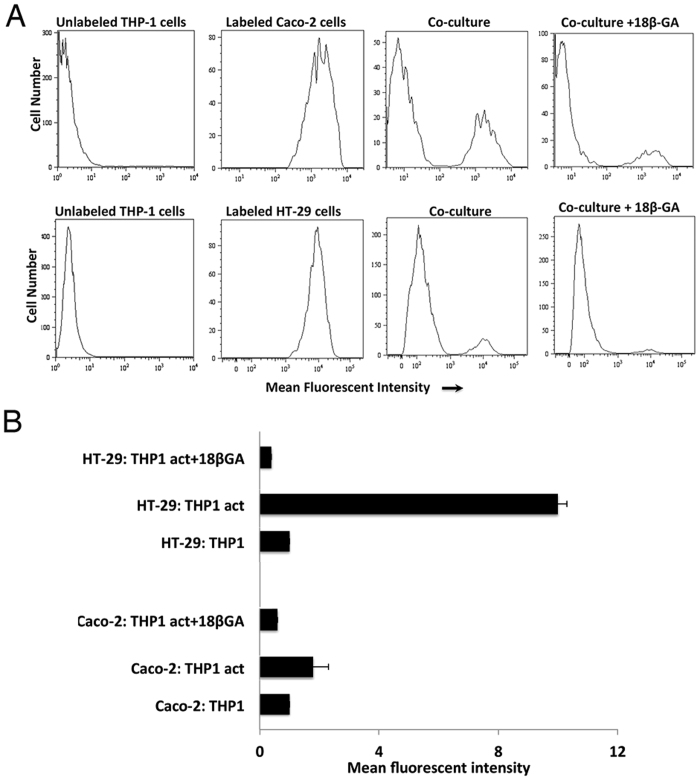
Hetero-cellular gap junction intercellular communication (GJIC) established between IECs and THP-1 cells. (**A**) Hetero-cellular gap junctional intercellular communication established between IECs and THP-1 cells. (**B**) Histogram represents the flow analysis of three independent experiments. The results are presented as the means ± S.E. of three independent experiments (One-way ANOVA with Tukey correction for multiple comparisons, p < 0.0001).

We showed a 2-fold increase in mean fluorescence intensity in Caco-2 co-cultured with THP-1 activated cells and a significant 10-fold increase in HT-29 co-cultured with THP-1 activated cells as compared to co-cultures with non-activated THP-1 cells (one-way ANOVA with Tukey correction p < 0.0001; Fig. 3B). The increase in dye coupling between IECs cultured onto activated THP-1 cells is supported by the increase in Cx expression in activated THP-1 cells (Fig. 2A). The formation of functional gap junction between IECs and THP-1 was further confirmed by addition of 18 β-GA; resulting in the reduction of dye transfer between these two cell types. Further, addition of a pannexin inhibitor, probenecid, to the IECs: THP-1 co-cultures did not affect the dye coupling between these two cell types (data not shown), emphasizing that the dye transfer occurred through gap junctions.

### Altered connexin expression in IBD tissues and possible communication between IECs and macrophages *in vivo*

**Figure 4:**
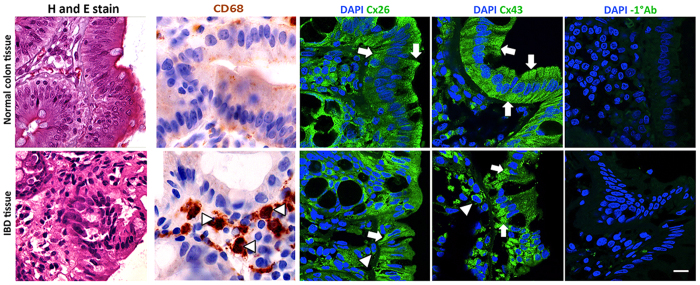
Connexin and CD68 expression in colon tissues. The arrows indicate the expression of Cx26 and Cx43 in epithelial cells in normal (upper panels), and in IBD tissues (lower panel). The arrowheads indicate macrophages. Negative control tissues (with exclusion of the primary antibody) is included, scale bar 10 μm. Images captured at a 63x/1.46 Oil Plan-Apochromatic objective. Increase of CD68 expression in infiltrating macrophages in IBD tissues, indicated by arrowheads, is observed as compared to normal colons. Images captured at a 100x Oil objective.

The data obtained from IECs-macrophages co-cultures *in vitro*, were supported by the expression and localization of Cxs in human colon tissues. We screened for Cx26 and Cx43 expression by immunofluorescence in paraffin embedded tissue sections, and showed that both Cxs are expressed at the apical and basolateral surfaces of the epithelial columnar cells in the colonic mucosa (Fig. 4). However, their expression was diminished at the apical surface and their localization was redistributed to the basolateral surface of epithelial cells in IBD tissues (Fig. 4). Thus, we hypothesized that this re-localization of connexin expression in IBD tissue might facilitate the communication between IECs and infiltrating macrophages, which is mediated by the degradation of the basement membrane. By immunohistochemistry, we confirmed the infiltration of macrophages by the strong expression of CD68 positive cells, which are situated in close proximity to the epithelial cells in IBD tissues (Fig. 4, arrowheads). Further, we demonstrated the degradation of the basement membrane by investigating the expression of collagen IV as the major component of the extracellular matrix. Histological and immunofluorescence staining showed that its expression is decreased in IBD tissues as compared to normal tissues (Supplementary Fig. 1S). We supported these data by *in vitro* experiments where we assessed the expression and activity Matrix Metalloproteinase (MMPs) in IECs by gelatin zymography. We showed that both the expression and activity of MMP-9, but not MMP-2, is up regulated in both Caco-2 and HT-29 treated cells with conditioned media from activated THP-1 cells as compared to untreated IECs (Supplementary Fig. 2S).

### Cxs-Dendra2 expression is upregulated by treatment with activated THP-1 conditioned media

**Figure 5:**
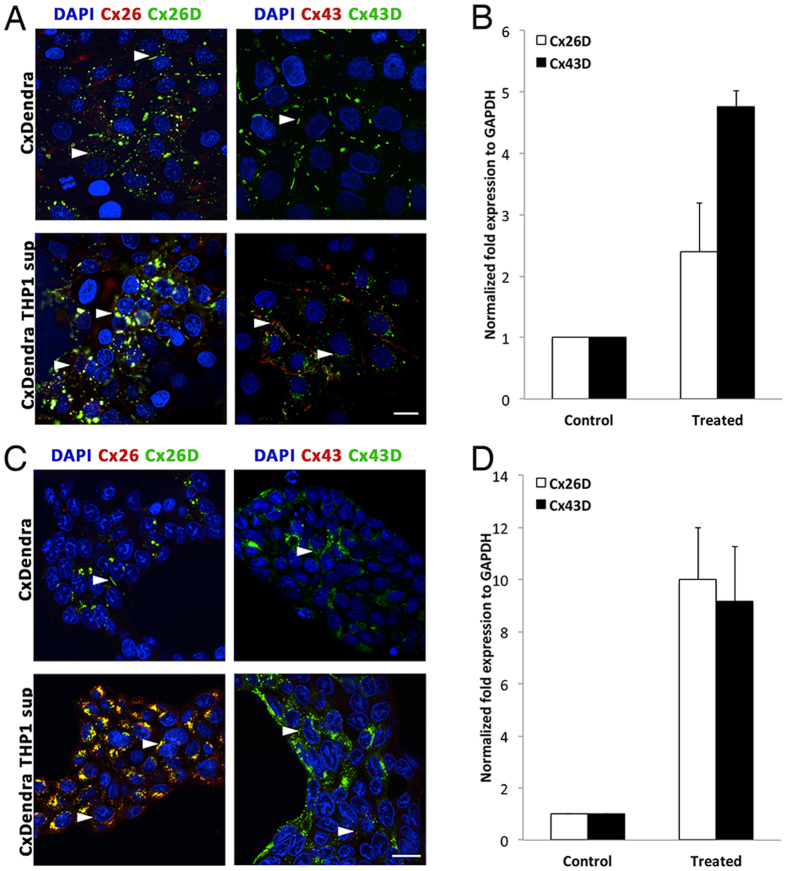
Cellular localization and expression of Cxs and Cxs-Dendra2 in IECs. (**A**,**C**) Expression of Cx26 (43) and Cx26 (43)-Dendra2 in Caco-2 cells and HT-29 cells, respectively. The arrowheads indicate the expression of Cx26 (43)-Dendra2 Scale bar 10 μm. Images captured at a 63x/1.46 Oil Plan-Apochromatic objective. (**B**,**D**). Densitometry analysis of western blots of Cxs-Dendra expression normalized to GAPDH in Caco-2 and HT-29 transduced cells, respectively. The results are presented as the means ± S.E. of three independent experiments ((**B**), for Cx43D p = 0.00015; (**D**), for Cx26D p = 0.016 and for Cx43D p = 0.02).

To study connexin expression and their localization with junctional complexes, we generated Cxs-Dendra2 lentiviral vectors. IECs were transduced with either Cx26-Dendra2 or Cx43-Dendra2 for 24 h and then treated with THP-1 conditioned media for an additional 24 h. The expression levels of Cxs-Dendra2 protein were studied by western blot. An increase in Cxs-Dendra2 expression was detected in both treated cell lines. A 2.5-fold and a significant 5-fold increase in Cx26-Dendra and Cx43-Dendra expression was detected in Caco-2 treated cells, respectively (p < 0.001; Fig. 5B). A significant 10-fold increase of both chimeras was determined in HT-29 transduced treated cells (p < 0.05; Fig. 5D).

In addition, we performed immunofluorescence assays to study the effect of conditioned media from activated THP-1 cells on cellular localization of endogenous Cxs and exogenous Cxs-Dendra2 chimeras. Cxs-Dendra2 transduced IECs were either untreated or treated with THP-1 conditioned media for 24 h. The cells were then fixed in ice-cold methanol at −20 °C, and immunostained for Cx26 and Cx43. In Fig. 5A, Caco-2 cells were successfully transduced with Cx26-Dendra2 and Cx43-Dendra2 chimeras, shown by the punctate dots around the nucleus and by the gap junction plaques between adjacent cells (arrowheads). Treatment of transduced cells with THP-1 conditioned media increased the expression of both endogenous Cx26 and Cx43 (red color) and exogenous Cx26 (43)-Dendra2 (green color). It is worth noting that exogenous Cx43 is more localized around the nucleus in treated Cx43 overexpressing Caco-2 cells, which is concomitant with increase expression of endogenous Cx43 forming GJ plaques. In Fig. 5C, Cx26 (43)-Dendra2 chimeras were observed in HT29 transduced cells and treatment with THP-1 conditioned media increased the expression of both endogenous Cx26 (43) and exogenous Cx26 (43)-Dendra2.

### Loss of junctional complex assembly in treated Cx43 overexpressing Caco-2 cells and in IBD tissues

**Figure 6:**
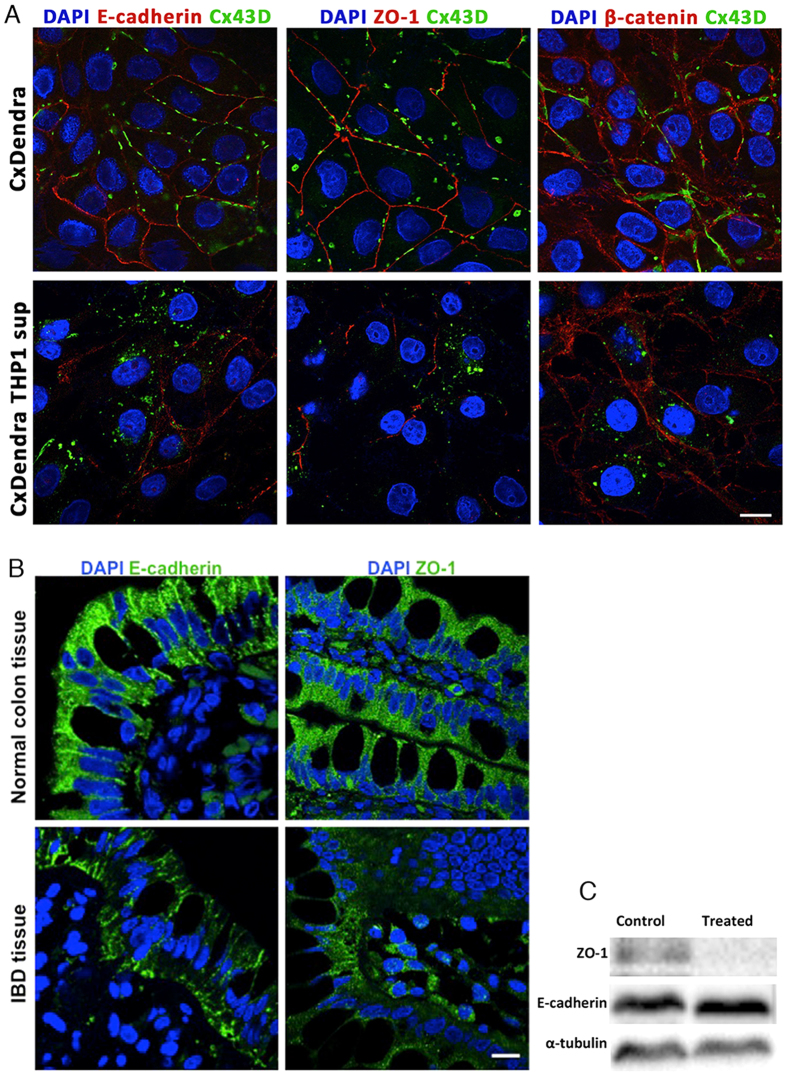
Expression of junctional complexes. (**A**) Co-localization images for E-cadherin, ZO-1, and β-catenin in Cx43 overexpressing Caco-2 cells. Scale bar 10 μm. Images captured at a 63x/1.46 Oil Plan-Apochromatic objective. (**B**) Expression of E-cadherin and ZO-1 in colon tissues. Scale bar 10 μm. Images captured at a 63x/1.46 Oil Plan-Apochromatic objective. (**C**) Protein expression of ZO-1 and E-cadherin in Cx43 overexpressing Caco-2 cells. α-tubulin used as internal control.

Alteration in the expression of adherens junction, α- and β-catenin, and tight junctions disrupts the epithelial barrier and increases paracellular permeability in IBD. To determine the effect of inflammatory mediators on junctional complexes assembly, immunostaining and western blot assays were performed. Connexin 43 was co-localized with E-cadherin, β-catenin, and ZO-1 at the intercellular junctions in Caco-2 cells overexpressing Cx43, as analyzed by LSM 710 software (Fig. 6A). Expression and co-localization of E-cadherin and ZO-1 was reduced in Caco-2 cells treated with THP-1 conditioned media (Fig. 6A). However, E-cadherin protein levels were not altered and ZO-1 expression was diminished in treated cells as compared to control cells (Fig. 6C). β-catenin expression was not altered and no translocation to the nucleus was detected in treated cells, hence, we did not study their expression at the translational level (Fig. 6A). In parallel, we showed by immunofluorescence that E-cadherin and ZO-1 expression in IBD tissues was decreased and the localization on the apical surface was altered as compared to normal colon tissues (Fig. 6B).

## Discussion

In intestinal inflammation, immune cells infiltrate the submucosa, juxtaposed to the epithelial lining of the intestine. This close proximity of immune cells to the epithelial cells suggests that a cross talk between these two cell types exists either directly through gap junctional channels or through soluble mediators and exosomes. Several studies have investigated this interaction in different tissues *in vitro.* Martin *et al*. showed that murine macrophages interact with intestinal epithelial cells through gap junctions, which provide means by which inflammatory cells might regulate IEC function^24^,^25^. Sharma *et al*. demonstrated a direct interaction between alveolar macrophages and alveolar epithelial cells that contributes to the initiation of acute pulmonary injury *via* a pro-inflammatory cascade^31^. Further, a study conducted by Khan *et al*. revealed that the direct interaction between HT-29 cells and tumor infiltrating leukocytes isolated from colorectal cancer increases the invasive properties of HT-29 cells^32^. Recently, Hyun *et al*. reported a crosstalk between IECs and macrophages mediated through TLR4, resulting in the production of IL-10 from human IECs that might regulate intestinal homeostasis^33^. However, few studies have reported the establishment of functional GJs between macrophage and epithelial cells and highlighted their role in inflammation^34^,^35^,^36^.

In the present study, we hypothesize that a hetero-cellular interaction between human IECs and macrophages plays an essential role in the regulation of intestinal epithelial barrier. We screened for the major connexins in two IECs (Caco-2 and HT-29) and in the monocytic/macrophage cell line (THP-1). We demonstrated that IECs and THP-1 cells express Cx26 and Cx43. We chose to focus our study on Cx26 and Cx43, for two main reasons: first, Cx26 and Cx43 have different trafficking pathways that allow for distinct interactions with connexin binding proteins, and second due to the demonstrated similarity between Cx43 and Cx45 channel characteristics. Earlier studies report the lack of Cx43 expression in HT-29 cells, and low expression of Cx26 and Cx43 in Caco-2 cells^37^,^38^,^39^. Studies have also shown the expression of Cx43 expression in monocytes^40^. We then demonstrated that connexins expressed in Caco-2 and HT-29 cells form functional homo-cellular GJ, and this direct cell-cell communication was reduced in the presence of GJ inhibitor as shown in dye transfer and FRAP assays.

We then explored the interaction between IECs and macrophages, by inducing an *in vitro* model for macrophages using the monocytic cell line THP-1 cells^28^,^29^,^30^. Although TLR2 gene expression was significantly up regulated in activated THP-1 cells, TLR4 gene expression was increased, however did not reach significant levels. We further showed that COX-2 and NF-κBp65 protein expression levels were up regulated in activated THP-1 cells. This activation induced the expression and the secretion of inflammatory cytokines such as IL-1β and TNF-α. These inflammatory cytokines increased the expression and the enzymatic activity of MMP-9 in treated IECs (Supplementary Fig. 2) presumably facilitating the breaching of the sub-epithelial basement membrane and bringing activated THP-1 cells in close proximity with IECs. Breaching of the basement membrane is associated with disruption of junctional complexes evident by the decrease in the protein expression of ZO-1 and E-cadherin junctional complexes (Fig. 6) and further supported by the morphological changes observed in IECs treated with inflammatory mediators (Supplementary Fig. 3). The increased activity of MMP-9 upon inflammatory stimulus is consistent with published data in IECs and colon tissues, implicating MMPs in the pathogenesis of IBD^41^,^42^. As a consequence, IECs adhere to THP-1 and a hetero-cellular communication is established. This communication was evident by dye transfer assays where a fluorescent calcein dye was transferred from labeled IECs to unlabeled THP-1 cells.

It is well established that the expression of receptor/ligand pair that mediates adhesion between two cells is required for the assembly of gap junction channels^43^,^44^. In this study, we proved that the dye coupling between IECs (Caco-2 or HT-29) and THP-1 activated cells was increased as the adhesion between these two cell types increased. This direct cell-cell communication was reduced in the presence of GJ inhibitor, but not affected in the presence of pannexin inhibitor, confirming that the dye transfer was through functional GJ channels. Further, the increase in dye coupling between IECs cultured onto activated THP-1 cells, mimicking the infiltrating macrophages in IBD, shown in this study is supported by the increase in Cx expression in activated THP-1 cells.

We supported our hypothesis of hetero-cellular communication between human IECs and macrophages by showing that connexin expression (Cx26 and Cx43) in IBD tissues is re-localized to the basolateral surface of epithelial cells as compared to normal tissue. We suggest that this redistribution of Cx expression facilitates the interaction of IECs with the infiltrated macrophages through formation of functional GJ. We supported our hypothesis by demonstrating a decrease of collagen deposition (Supplementary Fig. 1). and the strong expression of CD68 in infiltrating macrophage in IBD tissues.

We further investigated the effect of inflammatory mediators from activated macrophages on the expression of connexin and localization with junctional complex by overexpressing connexins in IECs. We showed that Cx43 was co-localized with E-cadherin, β-catenin, and ZO-1 at the intercellular junctions in Cx43 overexpressing Caco-2 cells. However, this association was lost under inflammatory conditions as assessed by immunofluorescence and western blot assays. We further examined the localization of E-cadherin and ZO-1 in IBD tissues and showed an alteration in the expression of both proteins. These results are consistent with earlier reports describing the loss of junctional complexes under inflammatory conditions in IBD patients^45^,^46^,^47^.

**Figure 7:**
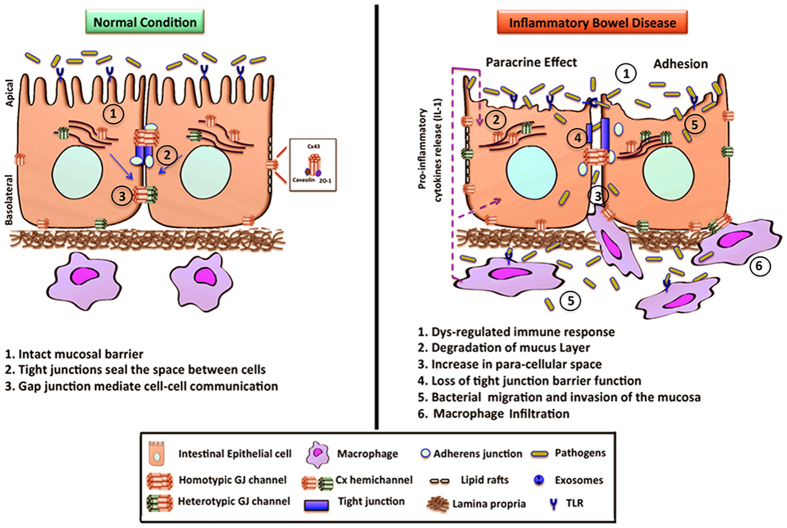
Proposed model for regulation of connexin in IBD.

In this study, we provide evidence that macrophages form functional gap junction channels with IECs. Connexin 26 and Cx43 expression, and MMP-9 enzymatic activity is upregulated under inflammatory conditions, while E-cadherin, ZO-1, and β-catenin expression is altered *in vitro* and *in vivo*. Collectively, we propose that the combination of paracrine and hetero-cellular communication between IECs and macrophages plays a pivotal role in the regulation of epithelial cell function by establishing junctional complexes between inflammatory cells and IECs that might contribute to the dysregulation of intestinal epithelial barrier as summarized by our model in Fig. 7. Further studies into the role of gap junctional communication in IBD can highlight the potential targeting of connexins as a therapeutic agent for this disease.

## Methods

### Cell lines and culture conditions

In this study, we used human intestinal epithelial cell lines (IECs): Caco-2 and HT-29 that are two colorectal adenocarcinoma cell lines widely used as models of intestinal transport and in pathology including inflammation. We also used a human non-adherent, monocytic cell line (THP-1), a human embryonic kidney cell line (293T) and a human cervical cancer cell line (HeLa).

THP-1 cells were used under two conditions: in suspension or activated with 50 ng/ml PMA for 24 h and with 1 μg/ml of LPS for additional 4 h to be utilized as adherent cells in the hetero-cellular communication assay. Following activation, the THP-1 cells were washed thoroughly to ensure no residual PMA and LPS in the media. The cells were then allowed to grow for 72 h with complete media. The supernatant were then collected 72 h post-treatment, filtered and used as conditioned media to treat IECs.

293T and HeLa cells were used for production of viral particles and for viral titration, respectively. Cells were maintained in RPMI 1640 (HT-29, THP-1), and DMEMAQ (Caco-2, 293T, HeLa). All media were supplemented with 10% FBS, 100 U/ml penicillin G and 100 μg/ml streptomycin. All cells were cultured at 37 °C in a humidified incubator with 5% CO_2_ atmosphere.

### Reagents

Cell culture media, fetal bovine serum (FBS), penicillin and streptomycin, trypsin, mouse anti-D-glyceraldehyde-3-phosphate dehydrogenase (GAPDH) antibody, phorbol 12-myristate 13-acetate (PMA), 18 β-glycyrrhetinic acid (18 β-GA), lipopolysaccharide (LPS), and Triton X-100 were purchased from Sigma (USA). Protein determination kit, N′, N′-bis-methylene acrylamide, polyvinylidene difluoride membranes, sodium dodecyl sulfate, glycine, Tris-HCl, TEMED, glycerol, ammonium persulfate, gelatin, and iQ SYBR GreenSupermix were from BioRad Laboratories (USA). NF-κBp65, β-catenin and α-tubulin antibodies, western blotting luminol reagents, and IgG horseradish peroxidase conjugated secondary antibodies were purchased from Santa Cruz Biotechnology (USA). Connexin 26, Cx43, and ZO-1 antibodies, Calcein-AM, goat anti-rabbit IgG conjugated Texas red, goat anti-rabbit IgG Alexa 488 and Prolong Anti-fade were from Life technologies (USA). COX-2 antibody was from Cayman chemical company (USA). E-cadherin antibody was from Cell Signaling Technology (USA). Mouse monoclonal antibody CD68 was from Leica Biosystems (UK). Protease and phosphatase inhibitors were from ROCHE (Switzerland). Restriction enzymes, T4 DNA ligase and gel band purification kit were purchased from MBI Fermentas (Canada). Endofree maxi plasmid purification kit was purchased from Qiagen (Germany). β-mercaptoethanol, agarose and ethidium bromide were from Amresco (USA). pDendra2-N plasmid and Dendra2 antibody were from Evrogen (Russia). Glass bottom culture dishes (Confocal dishes) were from MatTek Corporation (USA). LB agar and LB broth were from Difco Laboratories (USA). Normal goat serum (NGS) was from Chemicon (USA). Phusion Flash High-Fidelity PCR Master Mix and Revertaid 1st strand cDNA synthesis kit from Thermo Fisher Scientific (USA), Nucleospin RNA II kit from Macherey-Nagel (Germany), and Coomassie Brilliant Blue R-250 stain from Affymetrix (USA). Human IL-1β and TNF-α Quantikine ELISA Kits were purchased from R&D Systems (USA).

### Fluorescence recovery after photo bleaching (FRAP)

HT-29 cells, seeded on confocal dishes until confluent, were labeled with 1 μM calcein-AM for 1 h (LSM 710, Carl Zeiss, Germany). Pre-bleach images were taken. A specific cell was photo bleached at 10% 488 nm laser power for 5 iterations at 10 sec interval. Images were collected at 5 sec interval for a total of 8 min. Fluorescence intensity of the bleached cells was quantified and normalized to that of control cells.

### Dye Transfer Assays

IECs were seeded in 6-well plates at a density of 25 × 10^3^ per cm^2^ for 24 h. IECs were labeled with 1 μM calcein-AM for 1 h (HT-29) and 3 μM calcein-AM for 2 h (Caco-2), washed and incubated with serum-free medium for 30–60 min to allow intercellular esterase to convert non-fluorescent calcein-AM to a green-fluorescent calcein. Labeled IECs were co-cultured with either unlabeled IECs or unlabeled THP-1 cells for predetermined time intervals (as shown in Supplementary Fig. 4S). The co-cultures were incubated in complete media at 37 °C and 5% CO_2_ for 1 h. Non-adherent cells were then removed by washing and the adherent cells were detached by trypsinization and re-suspended in phosphate buffered saline (PBS) containing 2% formaldehyde to be analyzed by flow cytometry. Alternatively, co-cultures in suspension: THP-1: THP-1 and IECs: THP-1 were directly fixed in PBS containing 2% formaldehyde. Dye transfer was evaluated by measuring Mean fluorescence intensity (MFI) and data were calculated by dividing MFI over the percentage of adhered fluorescent cells after each co-culture. Mean fluorescence intensity of both the labeled and the unlabeled populations were determined by quadrant analysis of dot plots. Quadrant boundaries were set for each experiment based on fluorescence of unlabeled and calcein-labeled control samples. To confirm that calcein dye transfer was due to gap junction formation, cells were co-cultured in the presence of 100 μM of gap junction inhibitor, 18β-GA.

### Quantitative PCR

Total RNA was extracted from cells using Nucleospin RNA II kit as per manufacturer’s instructions. 1 μg of total RNA was reverse-transcribed to cDNA using Revertaid 1st strand cDNA synthesis kit. Quantitative PCR (qPCR) was performed using the iQ SYBR GreenSupermix in a CFX96 system (Bio-Rad Laboratories, USA). Products were amplified using primers that recognize Cxs, Toll-like receptors (TLR), MMPs, IL-1 β, TNF-α and GAPDH (Table 1). PCR parameters consist of a pre-cycle of 95 °C for 3 min followed by 40 cycles consisting of 95 °C for 10 sec, 52–62 °C for 30 sec, and 72 °C for 30 sec with a final extension at 72 °C for 5 min. The fluorescence threshold cycle value (Ct) was obtained for each gene and normalized to their corresponding GAPDH in the same sample. All experiments were carried out in duplicates and independently performed at least three times.

### Western Blot

Cells were washed with PBS and scraped at 4 °C in lysis buffer (0.5 M Tris-HCl buffer, pH 6.8; 2% SDS; and 20% glycerol). The samples were loaded onto 8–12% SDS-polyacrylamide gel, subjected to electrophoresis, and transferred to PVDF membrane. Following transfer, membranes were blocked with 5% skimmed milk and 0.05% Tween 20 in PBS (TPBS). Primary antibodies were then added for 3 h at RT or overnight at 4 °C. Blots were developed using horseradish peroxidase-conjugated secondary antibody and enhanced chemiluminescence detection kit. Positive and negative controls were performed to evaluate the specificity of the antibodies used. The intensity of bands, in the linear range of intensity, was quantified using ImageJ software (U. S. National Institutes of Health, USA).

### Immunofluorescence staining

IECs, untransduced or transduced with either Cx26-Dendra2 or Cx43-Dendra2, were cultured on coverslips, fixed with ice-cold methanol and stored at −20 °C. Cells were then washed 3X with PBS, and blocked with 5% NGS in PBS for 1 h in a humidified chamber. Cells were incubated with primary antibody for Cx26, Cx43, E-cadherin, β-catenin and ZO-1 antibodies for 2 h at RT, followed by washing and incubation with IgG-conjugated secondary antibody (Texas red: 1 μg/ml) for 1 h. The cells were then incubated with DAPI (1 μg/ml) for 10 min to stain their nuclei, washed 3X with PBS, mounted on slides using Prolong Anti-fade kit and observed by confocal microscopy.

Z-stacks of images were acquired using a 63x/1.46 Oil Plan-Apochromatic objective. Data analyses of co-localization and co-staining were performed using the Zeiss LSM 710 software.

### Immunofluorescence staining of paraffin-embedded tissues

Tissue sections were obtained from normal and IBD patients. All patients identifiers were kept confidential and no use of identities were utilized in this study. Biopsies referred to as “normal” were obtained from patients presented with abdominal pain; the diagnosed biopsies ruled out IBD. Tissues, 5-μm thick, were immunostained for Cx26, Cx43, E-cadherin and ZO-1 expression. Sections were heated to 50 °C for 40 min, washed 3X with xylol to remove the paraffin, and hydrated in a gradient series of alcohol to water. Antigen retrieval was performed by incubating sections in sodium citrate buffer (*p*H 6.0) and incubated at 100 °C for 40 min. Sections were allowed to cool for 15 min, washed 2X with deionized H_2_O and then were blocked with 5% NGS in PBS for 1 h in a humidified chamber. Sections were incubated with primary antibodies overnight at 4 °C, followed by washing and incubation with IgG-conjugated secondary antibody (Alexa 488: 1 μg/ml) for 1 h. The sections were then washed 2X with PBS, mounted with Prolong Anti-fade, and observed by confocal microscopy.

### Statistics

Statistical significance was determined by the unpaired Student’s t test. Error bar represents SEM of duplicates in three independent experiments. The p value was determined and values for p < 0.05 were considered significant. Differences between groups in Fig. 3B were assessed by one-way analysis of variance (ANOVA), followed by Tukey-Kramer HSD *post hoc* test.

## Additional Information

**How to cite this article**: Al-Ghadban, S. *et al*. Cross-talk between intestinal epithelial cells and immune cells in inflammatory bowel disease. *Sci. Rep.* **6**, 29783; doi: 10.1038/srep29783 (2016).

## Supplementary Material

Supplementary Information

## Figures and Tables

**Table 1:** Quantitative PCR primers.

Gene	Primer sequence	Annealing Temperature (^o^C)
Cx26	F: CCTCCCGACGCAGAGCAA	62
	R: CAGACAAAGTCGGCCTGCTCA	
Cx43	F: CTTCACTACTTTTAAGCAAAAGAG	52
	R: TCCCTCCAGCAGTTGAG	
TLR2	F: GGCTTCTCTGTCTTGTGACC	58
	R: GGGCTTGAACCAGGAAGACG	
TLR4	F: CCGCTTCCTGGTCTTATCAT	58
	R: TCTGCTGCAACTCATTTCAT	
TLR9	F: TACCAACATCCTGATGCTAGACTC	58
	R: TAGGACAACAGCAGATACTCCAGG	
MMP-2	F: TTGACGGTAAGGACGGACTC	55
	R: ACTTGCAGTACTCCCCATCG	
MMP-9	F: TTGACAGCGACAAGAAGTGG	55
	R: GCCATTCACGTCGTCCTTAT	
IL-1 β	F: TCCCCAGCCCTTTTGTTGA	60
	R: TTAGAACCA AATGTGGCCGTG	
TNF-α	F: AACATC CAACCTTCC CAAACG	58
	R: GACCCTAAGCCCCCAATTCTC	
GAPDH	F: TGGTGCTCAGTGTAGCCCAG	52–62
	R: GGACCTGACCTGCCGTCTAG	
