# Supplementary material for: Cross-talk between intestinal epithelial cells and immune cells in inflammatory bowel disease
Source: Sci Rep. 2016 Jul 15;6:29783. doi: 10.1038/srep29783 (PMC4945922; doi:10.1038/srep29783)
Supplement: Supplementary Information [file srep29783-s1.pdf]

Cross-talk between intestinal epithelial cells and immune cells in inflammatory bowel disease

Sara Al-Ghadban[1], Samira Kaissi[1], Fadia R. Homaidan[2], Hassan Y. Naim[3], and Marwan E. El-Sabban[1*]

[1]Department of Anatomy, Cell Biology, and Physiological Sciences, Faculty of Medicine,

[2]Inflammation group-Nature Conservation Center (NCC) for Sustainable Futures, American University of Beirut, Lebanon.

[3]Department of Physiological Chemistry, University of Veterinary Medicine Hannover, Hannover, Germany

*Corresponding Author: Dr. Marwan El-Sabban

E-mail: me00@aub.edu.lb

Running Title: GJIC between human IEC and MΦ

Supplementary methods

Construction of Cxs-Dendra2 chimeras

Cx26-Dendra2 and Cx43-Dendra2 chimeras were constructed by cloning connexins (Cxs) into the multiple cloning site (MCS) of pDendra2-N plasmid. Dendra2 is a monomeric green to red photo convertible fluorescent protein. Dendra2 was ligated to the C-terminal of the Cxs using Hind III and BamH1 restriction enzymes. cDNA covering the complete reading frame of Cx26 and Cx43 were synthesized from 1 µg of total cellular RNA and amplified by Phusion Flash High-Fidelity PCR Master Mix. PCR products were separated by electrophoresis on 1% agarose gel, and visualized with ethidium bromide staining. PCR fragments corresponding to Cx26-cDNA (780bp), Cx43-cDNA (1171bp), and pDendra2N (4700bp) were excised, purified, and digested with restriction enzymes. A ligation reaction containing three molar excess of the purified Cxs-cDNA to pDendra2-N was allowed to proceed for 20 min at room temperature (RT) using T4 DNA ligase. Aliquots of the ligation reaction were transformed into DH5α competent bacteria by heat shock. Positive colonies were identified by restriction enzymes analysis. New constructs within the plasmid were confirmed by sequencing (Applied Biosystems 3500 Genetic Analyzer, USA).

Transduction of IECs with Cxs-Dendra2 lentiviral vectors

For efficient delivery of Cxs-Dendra2 chimeric protein into IECs, we generated lentiviral vectors by cloning Cxs, N-terminally tagged with Dendra2 into the transfer vector pCSCW under the control of the CMV promoter. Using calcium phosphate, 293T cells were transfected with three lentiviral plasmids: pCMVDR8.91 (containing gag/pol),

pVSVG2 (containing the envelope gene VSV-G) and pCSCW-Cx-Dendra2. The cell supernatant containing the recombinant lentivirus was collected 48-72 h post transfection, filtered and frozen. To determine the viral titer, HeLa cells were transduced with the produced virus and the number of fluorescent cells was quantified by flow cytometry. The titer was extrapolated from the percentage of fluorescent cells, which correlates directly to the number of transducing viral units present in the supernatant used (tu/ml). A total of $2x10^6$ virus particles were used to transduce $2x10^5$ IECs. Forty-eight hours later, the efficiency of transduction was determined by the percentage of fluorescent cells on the plate. Transduced cells expressing the Cx-Dendra2 fusion proteins were used for biochemical studies.

Gelatin Zymography

50 μg of IECs proteins extracted from the cells or from the conditioned media of the cells were loaded into a stacking gel with 10% polyacrylamide resolving gel-containing gelatin. 2.5% FBS was used as a positive control to detect MMP-2 and MMP-9 enzymatic activities at 72 and 92 kDa, respectively. The gel was washed twice with a buffer containing Triton X-100 for 30 min and incubated overnight in a substrate buffer (1M Tris-HCl, pH 8; 0.07% $CaCl_2$ and 0.02% sodium azide) at 37 °C. The gel was then stained with 0.5% Coomassie Brilliant Blue R-250 stain for 1 h followed by a de-staining step using a de-staining buffer (30% ethanol, 10% acetic acid and 60% water) and visualized by Ultra-violet transilluminator (UPV company, UK); clear bands on a blue background indicate enzymatic activity.

Supplementary figures

1. Collagen expression in colon tissues

To demonstrate the degradation of the basement membrane, we investigated the expression of collagen in normal and IBD tissues by two methods: (1) Masson's trichrome stain where we showed that collagen fibers stained blue marks the basement membrane of the epithelium, however, in IBD tissues, collagen expand into lamina propria and submucosa. (2) Immunofluorescence assay where we determined that collagen expression is decreased in IBD tissues as compared to the normal tissues.

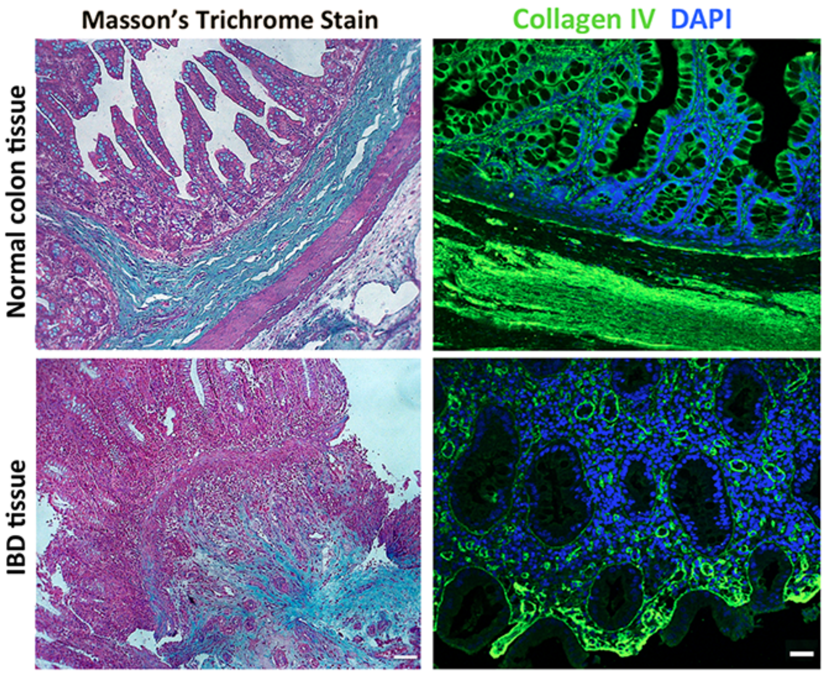

Supplementary Figure 1S - Collagen expression in normal and IBD tissues. Sections were stained with Masson trichrome stain for collagen fibers (Blue: Collagen, scale bar = 100 µm). For collagen type IV, scale bar: 50 µm. DAPI is a nuclear stain (blue).

2. Induction of matrix metalloproteinases in IECs under inflammatory conditions

To assess the activity of MMPs in IECs, gelatin zymography was performed. IECs treated with THP-1 supernatants increased both the expression and the secretion of MMP-9, but not MMP-2, as compared to control cells. An up regulation in protein expression of MMP-9 was observed in both cell lines, a 1.7-fold and a 2.5-fold increase in Caco-2 and HT-29 treated cells, respectively (Supplementary Fig. 2A). MMP-9 secreted levels were also upregulated by a 2.5-fold in Caco-2 treated cells and a 5-fold increase was detected in treated HT-29 cells as compared to untreated control cells (Supplementary Fig. 2B-D).

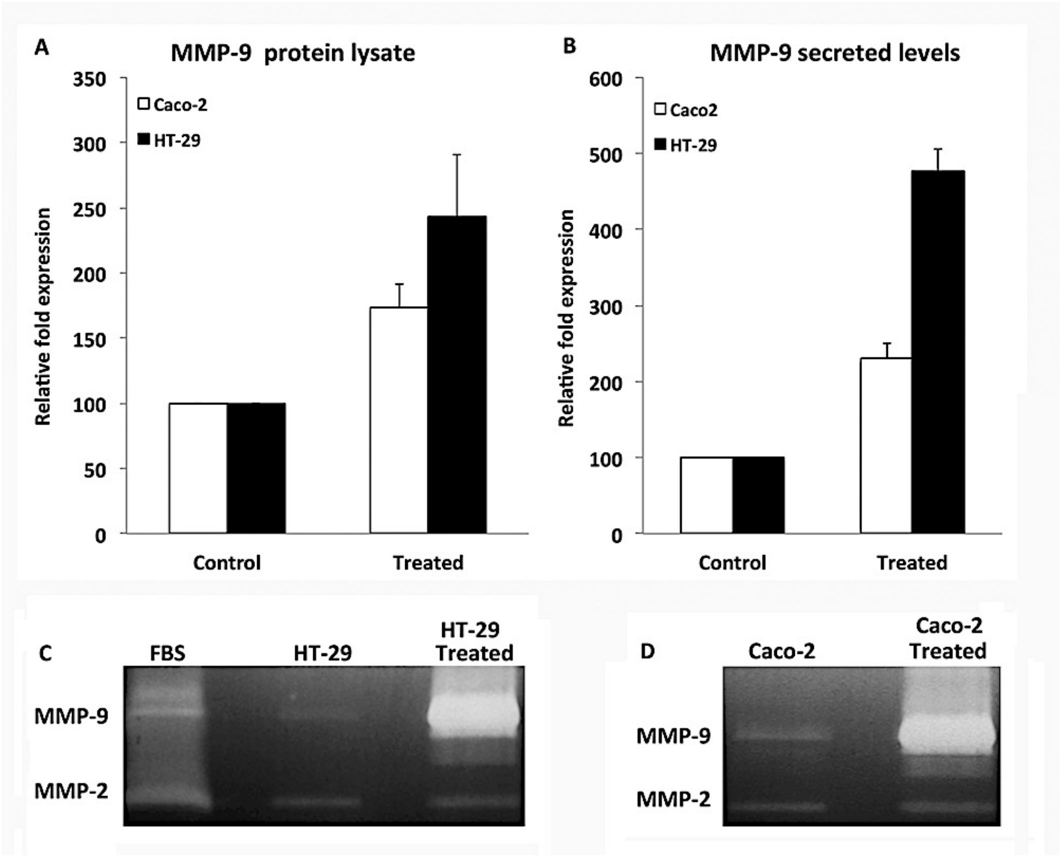

Supplementary Figure 2S - MMP-9 expression and secretion in IECs under inflammatory conditions. **(A, B)** Histogram analysis of zymogram gels of expressed and secreted MMP-9 in IECs. **(C, D)** Representative zymogram of secreted MMP-9 in IECs.

3. Cell Morphology

IECs cells were untreated or treated with conditioned media from activated THP-1 cells for 24h. Morphological changes in cells were observed following treatment (Supplementary Fig. 3).

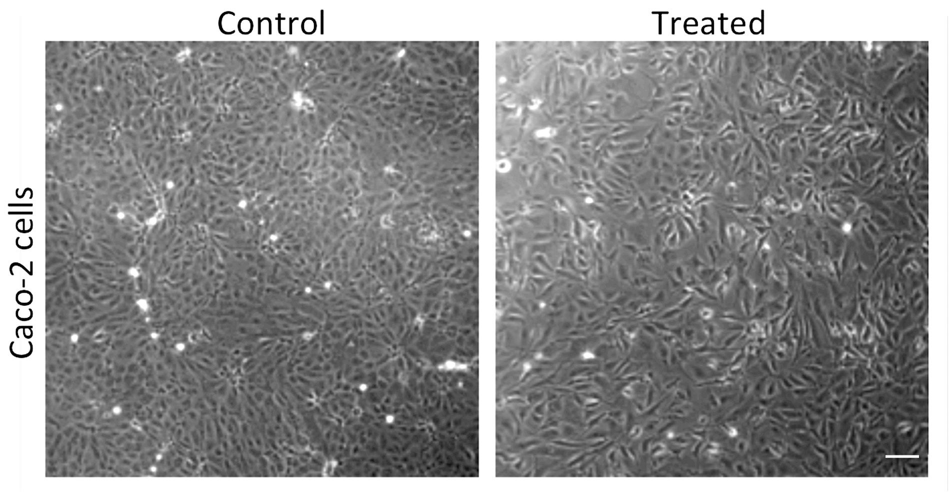

Supplementary Figure 3S - Bright field images of Caco-2 cells, control and treated with conditioned media. Scale bar= 100 µm.

4. Dye transfer assay

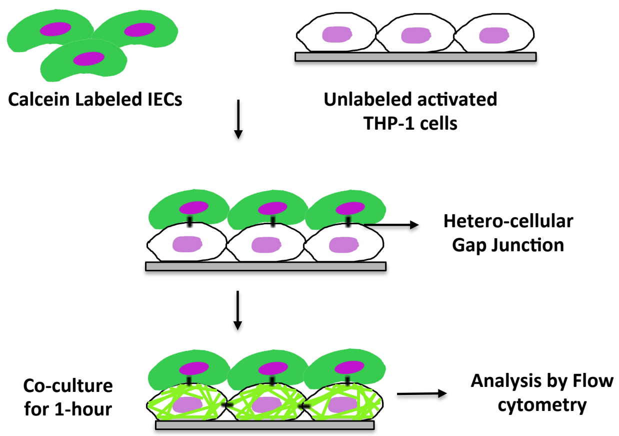

Supplementary Figure 4S - A schematic model for dye transfer assay.
